# Treatment of Low-Grade Bulbar Transitional Cell Carcinoma with Urethral Instillation of Mitomycin C

**DOI:** 10.1155/2008/173694

**Published:** 2008-10-28

**Authors:** Emmanuel J. Melonakos, Richard A. Santucci

**Affiliations:** ^1^School of Medicine, Wayne State University, Detroit, MI 48201, USA; ^2^Chief of Urology, Detroit Receiving Hospital, Wayne State University, Detroit, MI 48201, USA

## Abstract

A 63-year old man was referred to us after three rapid recurrences of low-grade urethral papillary transitional cell carcinoma of the bulbar urethra, after repeated primary excision. Cystoscopy confirmed 3-4 low-grade urethral transitional cell carcinomas, which were subsequently fulgurated. After urethral healing, a solution of Mitomycin C (40 mg/80 cc) was instilled into the urethra for fifteen minutes and held in place with a penile clamp. Urethral instillations were repeated weekly for six weeks. The patient is currently disease-free more than one year and three months posttreatment. This case highlights the successful treatment of urethral carcinoma with topical chemotherapy, which is usually reserved for the bladder, using a slight modification of standard technique.

## 1. INTRODUCTION

Cancer in the male urethra is rare, representing less than 0.5% of all malignancies in males [1, 2]. It is seen primarily in the sixth decade of life, with approximately 55–65% of tumors located in the bulbar part of the urethra and 30–35% occur in the anterior urethra [3]. Prognoses are not good for patients with advanced stages of bulbar urethral cancer, with only a 26% survival rate [4]. Bulbomembranous urethral tumors are typically diagnosed later than more anterior cancers, but prognosis is good if treated at early stages [4]. The chief problem in treating urethral carcinoma is how to expose the urethra to topical chemotherapy agents such as BCG or Mitomycin C. We present a case illustrating how to place chemotherapy effectively in the urethra, to curative effect in a patient with multiple previous failures of simple fulgaration.

## 2. CASE PRESENTATION

A 63-year old male was referred to our practice with recurrent, low-grade, bulbar urethra transition cell carcinoma which had recurred after two previous excisions.

The patient was taken to the operating room for a third excision, where several urethral tumors were removed. Pathology showed T1, Grade 1 urothelial carcinoma. A large caliber urethral stricture in the area was also treated with urethrotomy at the same time. After surgical excision, he underwent 6 weekly installations of 40 mg Mitomycin C reconstituted in 80 cc of saline. While the usual method of intravesical chemotherapy involves installation through a bladder catheter, in this case we used a catheter-tip “Toomey” syringe (Bard; Covington, Ga, USA) to gently instill the medication into the urethral meatus, then used a penile clamp (Storz; Culver City, Calif, USA) to keep the column of medication in the urethra for 15 minutes.

The patient suffered usual side effects of Mitomycin, including self-limited fatigue, dysuria, daytime urinary frequency up to every hour, and nocturia 2-3 X nightly [5]. All symptoms but nocturia resolved by the 6th treatment. He had cystoscopy and voided cytology examinations at 2, 6, and 12 and 18 months without recurrence of stricture or development of urethral cancer recurrences. (Previous recurrences X3 had been evident within 3 months of resection). The urethra appeared largely pink and healthy, with only small areas of white scar tissue marking the areas of previous urethral tumor resection.

## 3. DISCUSSION

**Intraurethral Mitomycin C is possible**

We report the successful application of Mitomycin C to urethral carcinoma, by a method of intraurethral installation and penile clamping to keep the agent in urethral contact. Local side effects included irritative voiding symptoms, but seemingly no more severe than after intravesical administration. Other authors have described significant complications including urethral stricture after Mitomycin was mixed into lidocaine jelly and placed into the urethra in one case. Penile and urethral complications of Mitomycin have also been reported after intravesical administration: (1) glans necrosis after high dose (60 mg) [6], (2) urethral sloughing, and (3) necrosis of the corpus spongiosum [7]. Despite these rare complications, practitioners wishing to treat urethral superficial transitional cell carcinoma with topical agents might consider this simple approach in their patients.

## 4. CONCLUSION

To our knowledge, this is the first case of bulbar, low-grade papillary transitional cell carcinoma treated successfully by application of liquid Mitomycin C. This technique provides a method for application of Mitomycin directly to the urethral mucosa.
